# Endometrial Stromal Sarcoma Arising in Colorectal Endometriosis: A Case Report and Review of the Literature

**DOI:** 10.1155/2015/534273

**Published:** 2015-01-12

**Authors:** Qiao Wang, Xia Zhao, Ping Han

**Affiliations:** ^1^Department of Gynecology & Obstetrics, West China Second Hospital, Sichuan University, Chengdu, Sichuan 610041, China; ^2^Department of Urology, West China Hospital, Sichuan University, Chengdu, Sichuan 610041, China

## Abstract

Extrauterine endometrial stromal sarcoma (ESS) arising in endometriosis is extremely rare, particularly in the colorectum. It should always be included in the differential diagnosis of primary tumors originating from gastrointestinal tract in females, given that preoperative endoscopical biopsy may reveal no specific changes. We reported a case of ESS arising in colorectal endometriosis and reviewed the previous 7 cases reported in the English literature. Our patient, who was unavailable for tumor resection and refused further adjuvant therapy, played a role in representing the natural history of low-grade extragenital ESS. This case was the only death from ESS arising in colorectal endometriosis.

## 1. Introduction

Endometriosis is a common gynecologic condition defined as an ectopic localization of endometrial tissue. Intestinal involvement of endometriosis occurs in 3% to 37% of all cases, most commonly involving the rectum and sigmoid colon [1, 2]. Malignant transformation of endometriosis is rare, occurring in 0.7% to 1% of all cases [3]. Most of the malignant neoplasms are endometrioid adenocarcinomas and clear-cell carcinomas [3]. Extrauterine endometrial stromal sarcoma (ESS) arising in endometriosis is extremely rare, particularly in the colorectum [4]. Traditionally, ESS was classified into low-grade and high-grade based on differences in mitotic activity, which is less than ten MF/10 HPF for low-grade ESS and ten or more MF/10 HPF for high-grade ESS [2].

In this paper, we presented a case of low-grade ESS arising in colorectal endometriosis and reviewed the previous 7 cases reported in the English literature. Up to date, our case reported the only death associated with ESS arising in colorectal endometriosis.

## 2. Case Presentation

**Figure 1:**
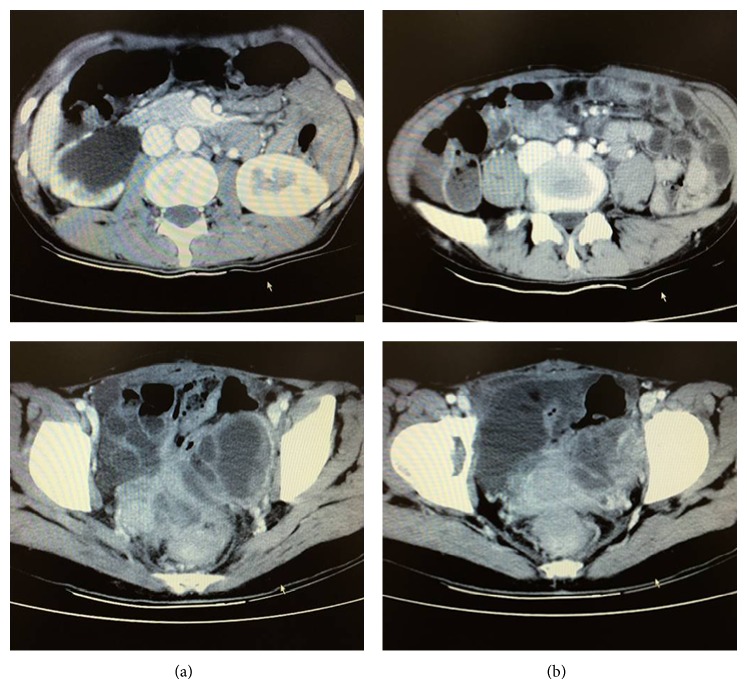
Abdominal and pelvic computed tomographic scan demonstrated thickening of rectal wall with inhomogeneous enhancement and a 4 cm soft tissue mass that involved the rectum and left ovary. Right hydronephrosis and hydroureter were also revealed.

A 40-year-old, gravida 3, para 2, woman presented with change in bowel habits and bright red rectal bleeding which had lasted for 1 year. She had a history of subtotal abdominal hysterectomy for uterine leiomyoma performed 3 years before and a history of right ovarian cystectomy. She denied any symptoms or presentations of endometriosis and had no history of hormone replacement therapy. Digital rectal examination revealed a fixed and firm mass with blood. Her serum carcinoembryonic antigen levels were in normal limits. The laboratory tests showed no remarkable abnormality except for a moderate anemia. Abdominal and pelvic computed tomographic (CT) scan demonstrated thickening of rectal wall with inhomogeneous enhancement and a 4 cm soft tissue mass that involved the rectum and left ovary (Figure 1). The CT scan also revealed right hydronephrosis and hydroureter. On colonoscopic examination, a protruding lesion in the rectum and segmental stenoses of the rectosigmoid lumen were found. Endoscopic biopsy reported tubular adenoma and proliferation of spindle cells at the edge of tissue. Laparotomy was performed under the clinical consideration of gastrointestinal stromal tumor (GIST) or carcinoma developed on the rectum. On operation, nodular masses (1–3 cm in diameter) were found generally scattered in the intestinal walls and mesentery. Frozen section examination of nodules reported the exhibition of endometrial glands and whirling proliferation of plump spindle cells, considering extrauterine ESS as a clinicopathologic diagnosis. Because of the extensive intra-abdominal metastases and adhesion, the lesion was unresectable. The patient underwent intraoperative peritoneal chemotherapy and palliative transverse colostomy to relieve the stenosis. On immunohistochemical stain, the tumor cells were diffusely positive for CD 10, estrogen, and progesterone receptors (and 7% positive for Ki-67) but negative for CD 117, *α*-inhibin, desmin, smooth muscle actin, caldesmon, CD 34, and S-100. The diagnosis of low-grade ESS was made. After surgery, the patient refused further chemotherapy or radiotherapy. She died associated with this disease 18 months after diagnosis.

## 3. Discussion

In 1925, Sampson first described and suggested 3 criteria for diagnosis of malignancy arising in endometriosis: (1) close proximity of benign endometriosis to the malignant tumor, (2) no other primary site identified, and (3) tumor histology compatible with an endometrial primary [5]. The ovary is the primary site in 76% of the cases, whereas the colorectum is involved in only 5% of cases [3, 4]. ESS is a very uncommon histopathologic type of malignancy in endometriosis. We searched MEDLINE database and identified a total of 87 cases of ESS arising in endometriosis reported before August 2014. Together with the data of our present case, the most commonly involved areas included ovary (37/87, 42%), pelvis (18/87, 20.5%), and colorectum (8/87, 9.2%).

We reviewed the current 8 cases (including our present one) of colorectal ESS arising in endometriosis [6–11]. The average age of the 8 cases was 55 (range, 38–63) years. Five cases were Caucasian, and the other 3 were Asian. The common clinical complaints were abdominal/pelvic pain (4/8, 50%), change in bowel habits (2/8, 25%), difficult defecation (2/8, 25%), and rectal bleeding (2/8, 25%). Rectum was the most commonly involved site, followed by sigmoid colon. Four cases had a history of endometriosis. Three cases had a history of gynecologic surgery for benign diseases. Two cases had ever received replacement hormone therapy. Only one case of low-grade ESS which occurred in colorectum was reported [10]. Five cases were positive for metastasis. One case reported by Yantiss underwent a recurrence of tumor 3 years after tumor resection but is alive and disease-free 6 years after her initial diagnosis, following adjuvant radiotherapy [7]. The reported complications included portal vein thrombosis [1] and disseminated intravascular coagulation [9]. Our present case was the only one unavailable for tumor resection and dead of disease. The clinical characteristics, management, and outcome of each case were demonstrated in Table 1.

ESS arising in colorectal endometriosis should always be included in the differential diagnosis of primary tumors originating from gastrointestinal tract in female patients. Endoscopically, the intestinal mucosa frequently shows minimal changes. Endoscopical biopsy may reveal only nonspecific changes. Only one case in our review had been correctly diagnosed via endoscopical biopsy before surgery. GIST is easy to be confused with ESS because of variable gross and histological appearance. Immunohistochemical stains are useful to distinguish these two entities. GIST is known to stain diffusely for CD 117 and CD 34, while ESS is negative for CD 117 and CD 34 but frequently positive for CD 10 and estrogen or progesterone receptors [9, 12].

Given the rarity of these tumors, evidence-based data are not available to help guide treatment decisions. Surgery is generally regarded as the cornerstone of treatment for ESS. Complete cytoreductive surgery is recommended, especially in patients for whom this surgery could result in being residual-disease-free. Tumor-free margins are important in prognosis [12]. However, the effort of total hysterectomy and bilateral salpingooophorectomy in extragenital ESS remains unproven. Extragenital ESS may have a high tendency for dissemination and metastasis in the omentum, mesentery, and abdominal or pelvic wall [13], which is correspondent with the finding in our present case. Despite this, most patients with extrauterine ESS had prolonged disease-free intervals with late recurrences [13]. For patients of peritoneal dissemination, cytoreductive surgery should still be attempted given the fact that low-grade tumors are amenable to hormone receptor-targeted therapy which would lower the risk of recurrence. The value of adjuvant therapy is controversial with no prospective studies showing a survival advantage associated with the use of chemotherapy or radiotherapy. Nevertheless, adjuvant therapy is considered in patients with metastatic or recurrent ESS. Radiotherapy may also serve a palliative role to manage pain, bleeding, and compression of surrounding organs [12]. The prognostic predictor of ESS is unclear. It was reported that the prognosis of high-grade ESS was worse than low-grade ESS [14]. A lifelong follow-up is necessary.

In our case, the patient was unavailable for tumor resection and refused further adjuvant therapy. Thus, the outcome could represent the natural history of extragenital ESS. Moreover, this case was the only death from low-grade ESS arising in colorectal endometriosis.

In conclusion, we presented a rare case of disseminated ESS arising in colorectal endometriosis, with a nature of poor prognosis. Even though malignant transformation of extragenital endometriosis is extremely rare, it should be included in the differential diagnosis of colorectal tumors in women.

## Supplementary Material

HE stain of resected nodules reported the exhibition of endometrial glands and whirling proliferation of plump spindle cells, considering extrauterine ESS as a clinicopathologic diagnosis.

## Figures and Tables

**Table 1:** The clinical characteristics, managements, and outcomes of cases reported as colorectal ESS arising in endometriosis.

Author, year	Age (year)	Race	Parity	Symptoms	History of gynecologic surgery	History of endometriosis	History of hormone therapy	Site of mass	Histopathologic type	Metastasis	Management	Follow-up, months
Baiocchi et al., 1990 [6]	38	Caucasian	0	Abdominal pain and pressure	TAH, BSO	Yes	Yes	Colon	Low-grade ESS	Mesentery and pelvis	TR, CT, HT	NED, 16
Yantiss et al., 2000 [7]	63	Caucasian	NA	Change in bowel habits	NA	No	No	Rectum	Low-grade ESS	NA	TR, RT	NED, 60
Bosincu et al., 2001 [8]	42	Caucasian	NA	Abdominal pain and fever	NA	Yes	No	Rectum	Low-grade ESS	Omentum and pelvis	TAH, BSO, TR, CT	NED, 20
Mourra et al., 2001 [1]	61	Caucasian	1	Abdominal pain	NA	No	Yes	Rectum	Low-grade ESS	No	TR	NED, 30
Cho et al., 2002 [9]	48	Asian	NA	Difficult defecation and tenesmus	TAH, BSO	Yes	NA	Rectosigmoid colon	Low-grade ESS	Mesentery and urinary tract	TR	NED, 4
Chen et al., 2007 [10]	42	Asian	Multiple	Difficult defecation and rectal bleeding	No	No	No	Rectosigmoid colon	Low-grade ESS	Omentum and left ovary	TAH, BSO, TR, RT	NED, 12
Roşca et al., 2011 [11]	51	Caucasian	NA	Pelvic pain	NA	Yes	No	Rectosigmoid colon	Low-grade ESS	NA	NA	NA
Present	40	Asian	2	Change in bowel habits and rectal bleeding	TAH	No	No	Rectum	Low-grade ESS	Mesentery and extensive intra-abdominal metastases	Palliative surgery	DOD, 18

TR: tumor resection; CT: chemotherapy; RT: radiotherapy; HT: hormone therapy; TAH: total abdominal hysterectomy; BSO: bilateral salpingooophorectomy; NED: no evidence of disease; DOD: dead of disease; NA: not available.
