# Supplementary figures and images for: Endometrial Stromal Sarcoma Arising in Colorectal Endometriosis: A Case Report and Review of the Literature
Source: Case Rep Obstet Gynecol. 2015 Jan 12;2015:534273. doi: 10.1155/2015/534273 (PMC4306220; doi:10.1155/2015/534273)

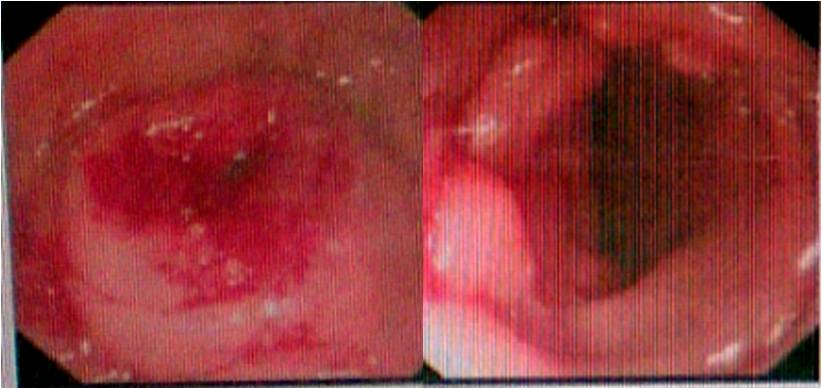

Supplement: Supplementary file 1 — HE stain of resected nodules reported the exhibition of endometrial glands and whirling proliferation of plump spindle cells, considering extrauterine ESS as a clinicopathologic diagnosis. [file 534273.f1.jpg]
